# Four Channel Multivariate Coherence Training: Development and Evidence in Support of a New Form of Neurofeedback

**DOI:** 10.3389/fnins.2018.00729

**Published:** 2018-10-11

**Authors:** Robert Coben, Morgan Middlebrooks, Howard Lightstone, Madeleine Corbell

**Affiliations:** ^1^Integrated Neuroscience Services, Fayetteville, AR, United States; ^2^EEG Software, LLC, Gainesville, FL, United States; ^3^Department of Psychological Science, University of Arkansas, Fayetteville, AR, United States

**Keywords:** neurofeedback, QEEG, neuromodulation, coherence, connectivity, EEG

## Abstract

As the field of neurofeedback and neuromodulation grows, trends toward using neurofeedback to treat problems of brain dysfunction have emerged. While the use of connectivity based fMRI guided neurofeedback has shown itself to be efficacious, the expense related to the treatment calls for a more practical solution. The use of QEEG guided neurofeedback in the treatment has shown promise as an emerging treatment. To date, EEG based neurofeedback approaches have used technology with limited sophistication. We designed a new form of neurofeedback that uses four channels of EEG with a multivariate calculation of coherence metrics. Following a mathematical presentation of this model, we present findings of a multi-site study with clinical subjects with various diagnoses. We compared this form of multivariate coherence neurofeedback to the more standard two channel coherence training. Findings showed that there was a significant difference between the groups with four channel multivariate coherence neurofeedback leading to greater changes in EEG metrics. Compared to two channel coherence training, four channel multivariate coherence neurofeedback led to a greater than 50% change in power and 400% in coherence values per session. The significance of these findings is discussed in relation to complex calculations of effective connectivity and how this might lead to even greater enhancements in neurofeedback efficacy.

## Introduction

To find solutions to neuropsychiatric problems attributed to the disruption of brain function, a trend has developed toward using different modalities of neuromodulation and brain computer interface (BCI). Different forms of neuromodulation have shown efficacy as potential interventions in clinical populations. Sun et al. (2017) showed that BCI could help participants regain motor function following stroke. Rota et al. (2009) showed that deep transcranial magnetic stimulation (dTMS) was an effective treatment method for 212 participants experiencing major depressive disorder who were non-responsive to antidepressant medication. In a study by Sokunbi et al. (2014), participants who were exposed to real time functional magnetic resonance imaging (fMRI) signal and a visual feedback system were able to reduce activation in areas of the brain associated with the motivational processes of addiction in 8 out of 10 instances. Rota et al. (2009) employed the use of fMRI-neurofeedback to improve participants’ performance on linguistic tasks including improvement in the identification of emotional prosodic intonations.

Megumi et al. (2015) noted that participants who underwent connectivity based fMRI neurofeedback training to improve connectivity between two target regions of interest in the brain maintained these improvements 2 months after training had ceased, indicating that connectivity based fMRI neurofeedback can provide lasting changes. While the use of fMRI based neuromodulation has shown efficacy as an alternative form of treatment, the use of fMRI is not cost effective or practical and yields low temporal resolution (Frey et al., 2013). Alternatively, electroencephalography (EEG) is relatively inexpensive, practical, and yields high temporal resolution (Frey et al., 2013). For these reasons, EEG is a practical neuromodulation technique for BCI procedures, and could potentially impact a greater number of people in need.

EEG based neurofeedback therapy has demonstrated efficacy in the treatment of clinical conditions and symptoms including decreasing symptoms related to attention deficit hyperactivity disorder (ADHD) (Arns et al., 2009; Moriyama et al., 2012), seizure disorders (Sterman and Egner, 2006), and learning disabilities (Fernandez et al., 2003; Coben et al., 2015). Additional findings have shown that EEG based neurofeedback can reduce symptoms associated with autism spectrum disorder (ASD) (Kouijzer et al., 2009; Coben, 2013). Moreover, a 2016 study found that EEG characteristics associated with autism were reduced using prefrontal neurofeedback treatment (Wang et al., 2016). There is also evidence that the effects of these interventions last beyond the initial training period (Gevensleben et al., 2010; Coben, 2013).

Many researchers have found the use of two channel coherence training to be efficacious and a good alternative to traditional methods that are often more time consuming and expensive. In a 2002 study, participants with mild head injury were able to improve their self-reported symptoms using quantitative electroencephalography (QEEG) guided two channel coherence training in an average of 19 sessions (Walker et al., 2002). Two channel coherence training was shown in a 2015 randomized control trial to improve the reading scores of children when compared to children who attended traditional resource room style reading programs. The experimental group on average improved their reading scores by 1.2 grades levels in 10 weeks (Coben et al., 2015). Coben and Padolsky (2007) utilized two channel neurofeedback in the treatment of 37 children diagnosed with ASD to significantly decrease the presence of ASD symptoms by 40% in 20 sessions.

The commonly used two channel method is now understood to lead to erroneous findings and spurious flows. The growing body of research concerning coherence assessment suggests that using a greater number of electrodes relative to the standard two channel approach increases spatial acuity (Blinowska, 2011). As noted by Blinowska (2011), the lack of spatial acuity increases the likelihood of arriving at spurious findings. Previous research has cited error rates up to 50% when using bivariate coherence measures. A lack of spatial acuity is largely responsible for the high error rate (Black et al., 2008). These findings identify the need for the development of more effective neurofeedback techniques compared to the two channel coherence approach.

We hypothesize that four channel multivariate coherence neurofeedback training may increase efficacy to improve upon the existing two channel form of coherence neurofeedback training. Utilizing multiple channels as opposed to the standard two channels allows for a larger number of comparisons. A single frequency band contains 171 possible comparisons, many of which do not correspond with known neural pathways. Additionally, comparisons that use two channels are not as precise because they assume 2-dimensional space rather than 3-dimensional space. (Coben et al., 2014b). Multiple channel coherence is advantageous because of its higher level of precision compared to standard pairwise coherence (Coben et al., 2014a).

## Development of Four Channel Multivariate Coherence Training

As a practical solution to the problems that arise when using two channel coherence training, we developed a version using four channels that measure coherence in a multivariate fashion between all possible combinations of electrodes.

This led to the use of EEGer4, a software component of a neurofeedback system. With a supported amplifier and electrodes, EEGer becomes a complete neurofeedback tool (EEG Software, 2013). EEGer works by receiving voltage samples from a supported amplifier at 256 Hz (recording the samples), and processing the data in several different ways. The simplest process is just to digitally filter the incoming data and compare the amplitudes of the filtered bands of data to some threshold value. Only when all signals are in a correct state is a “reward” granted. This reward can be visual or audible. This is the simple process used in most neurofeedback systems.

EEGer filters are all elliptical infinite impulse response (IIR) filters (Double precision digital elliptic filter design program by Gray and Markel, IEEE T-ASSP vol. 24, no. 6, Dec. 76).

Each IIR filter is comprised of a number of “stages” where each stage applies further “sharpness” to the frequency limits of the data passing through the filter. Low-pass filters in EEGer are one-stage filters while band-pass filters are 2-stages or more. EEGer receives the incoming data and filters the data to remove 50 Hz and above data (usually powerline noise). The incoming low-pass filter provides a low delay in the incoming signal while removing high frequency noise. The filtered (low pass) data is then broken down into narrower filtered data bands (using IIR filters of 2 stages). The incoming data is delayed by three stages (stage 1 is the low-pass filter, and stages 2 and 3 are the band-pass filter; sample times). The analysis model used for this coherence measure has several steps in operation. The first step is called “PSync” (phase synchrony) in the EEGer filter documentation.

The PSync calculation uses inputs from two channels of data. The filter band data from each channel (with the same frequency limits) is kept in a “history” buffer for each channel. The (time) length of the buffer is based on the upper frequency value of the filtered band. The two input streams yield two values (X and Y). A cross correlation is then performed on a window (W) of the value histories:

(1) $$\frac{\int X(t)Y(t)}{\sqrt{\int X(t)X(t)\int Y(t)Y(t)}}$$

This reduces in practice to:

(2) $$\frac{\sum_{1}^{W}X(t)Y(t)}{\sqrt{\sum_{1}^{W}X(t)X(t)*\sum_{1}^{W}Y(t)Y(t)}}$$

*X(t)* is the summation of the absolute amplitude of the history values of one channel.

The window width defaults to 0.5 s. The resulting correlation value is smoothed using a standard externally weighted moving average (EWMA) filter. EWMA is calculated using a portion of the previous running value and part of the newest value. This standard statistical method is also called a “rolling mean” or “moving mean” where the exponential factor is based on the sample rate of the data. This value ranges from 0 to 1.

**FIGURE 1:**
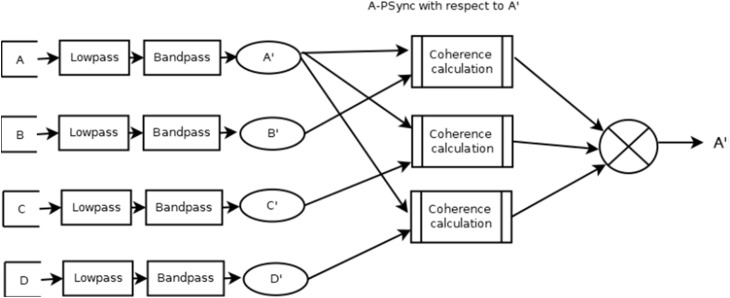
Data flow of QPSavg mode.

There is another filter mode named A-PSync where a PSync operation is performed on one or more pairs of input channels (a Psync computation on each pair). The average value of the (up to three) Psync operations is the output. There may be less than three values used in the average. Each separate value is compared against its threshold value and only used if the value is in a “rewardable state.” A “rewardable state” can be a number of different measures but is typically either signal-above-threshold or signal-below-threshold. Again, if this bare output is being used, it is EWMA-smoothed over a user-selectable value (default is 0.5 s). **Figure 1** is a pictorial representation of the A PSync filter.

The QPS filter models use multiple copies of the A-PSync filter. Up to four A-PSync (each of which can be an average of coherence of some channels with respect to one channel) inputs can be combined in a number of ways. The combinations are named QPSavg, QPSlag, and QPSdev. The resulting operation depends on the submodes described below.

QPSavg: The values are summed and divided by the number of streams.

QPSlag: The values are integrated using an EWMA filter that is delayed in time compared to the base of QPSavg.

QPSdev: The deviation of the values is computed.

The resulting signal is smoothed over the user-specified time and then compared against a threshold value to determine if it is in a “rewardable state.”

**FIGURE 2:**
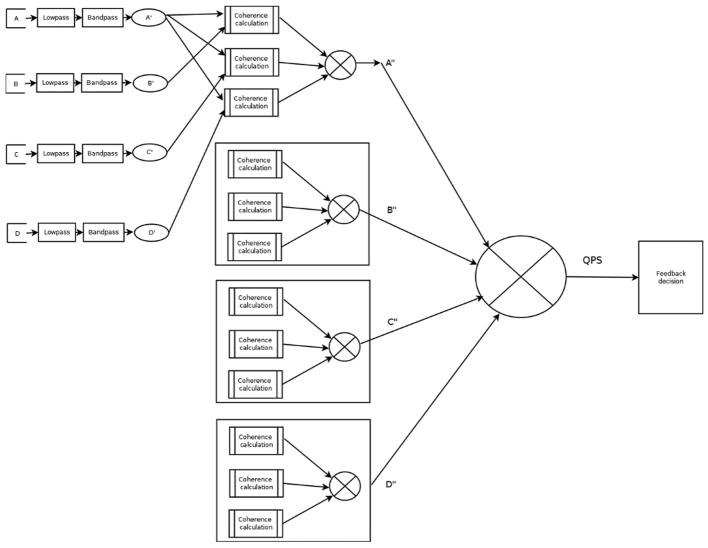
Visual representation of A_Psync filter.

**FIGURE 3:**
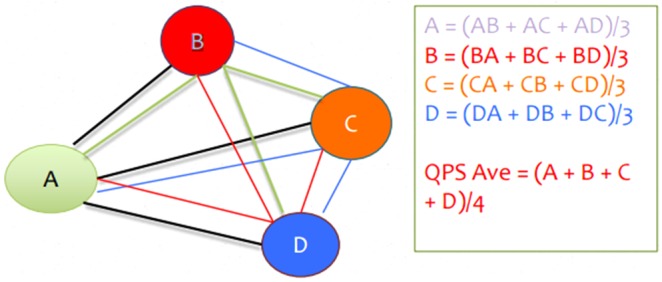
Graphical diagram of QPSavg calculation.

**Figure 2** presents a graphical diagram of the A_Psync mode. A graphical data flow diagram of the QPSavg mode is depicted in **Figure 3** in a simplified form.

In short, inputs from sensors are transformed into multivariate coherence values across all possible combinations of the four locations or sensors. This information makes up the reward band. Additionally, there are three user-defined inhibit hands that encourage a decrease in EEG frequencies, as a form of amplitude training. For example, you can decrease theta for those diagnosed with ADHD.

To investigate the idea that four channel multivariate coherence training will provide more efficacious results than two channel coherence training, we conducted an independent measures experimental study. The study compares the coherence and power change scores for a group of individuals who participated in either a four channel multivariate coherence training group or a two channel coherence training group. The type of neurofeedback treatment will serve as the independent variable, while the outcomes scores in terms of changes in coherence and power will serve as dependent measures of change.

## Materials and Methods

### Participants

During the study 174 participants were selected from 11 different sites in Colorado, New York, Ohio, New Jersey, Pennsylvania, and Florida. The contributing clinics and participants were volunteers and were not compensated from their contributions to the study. All participants or their parents understood the nature of their participation and provided written consent. They were divided into two groups; 61 participants received the standard two channel coherence training, while 113 of the participants received four channel multivariate coherence training. Participants in the four channel group were randomly assigned to either the QPS average, lag or deviation mode. The mode was assigned by the principal investigator, no clinic independently assigned participants to QPS groups. 39 participants received QPS average training, 40 participants received QPS deviation training, and 34 received QPS lag training.

The sample represented children and adults of various diagnoses. The participants had a range of diagnoses, as displayed in **Table 1**. The most frequent diagnoses were ASD (ASD = 30.46%, *n* = 53) and ADHD (ADHD = 16.67%, *n* = 29).

**Table 1:** Frequency of participant diagnoses.

Diagnosis	Frequency	%	Valid %	Cumulative %
ADHD	29	16.7	16.7	16.7
ADHD and LD	5	2.9	2.9	19.5
ADHD, LD, and anxiety disorder	1	0.6	0.6	20.1
Anoxia	1	0.6	0.6	20.7
Anxiety	3	1.7	1.7	22.4
APD	3	1.7	1.7	24.1
ASD	53	30.5	30.5	54.6
ASD and LD	4	2.3	2.3	56.9
Auditory processing disorder and LD	1	0.6	0.6	57.5
Bipolar disorder	1	0.6	0.6	58.0
Cognitive disorder	2	1.1	1.1	59.2
Cognitive disorder	8	4.6	4.6	63.8
Cognitive disorder and depression	2	1.1	1.1	64.9
Concussions	1	0.6	0.6	65.5
CVA	2	1.1	1.1	66.7
Depression	10	5.7	5.7	72.4
Developmental delays	2	1.1	1.1	73.6
Epilepsy	8	4.6	4.6	78.2
Language disorder and anxiety	1	0.6	0.6	78.7
Language disorder and LD	1	0.6	0.6	79.3
Learning disability	13	7.5	7.5	86.8
LD and depression	1	0.6	0.6	87.4
OCD	2	1.1	1.1	88.5
OCD and LD	1	0.6	0.6	89.1
Pervasive developmental disorder	2	1.1	1.1	90.2
Seizure disorder	1	0.6	0.6	90.8
TBI	15	8.6	8.6	99.4
Vascular dementia and depression	1	0.6	0.6	100.0
Total	174	100.0	100.0	

Participants ranged from 4 to 80 years old, with a mean age of 19.526 (*SD* = 18.115). Approximately two-thirds (64.9%) were male, while 35.1% were female. The vast majority of the participants were right-handed (88.82%), while the remainder were either left-handed (10.00%) or ambidextrous (2.30%). The majority (79.9%) were not using psychoactive medications, while 10.9% were using 1 medication, 3.4% were using two medications, and 4% were using three or more medications at the beginning of their training.

There were no significant differences for gender, handedness, age, medications, channels, or QPS modality across clinics. The only significant differences found were for diagnosis, such that different clinics tended to treat more of a certain problem than others. For example, some clinics saw significantly more participants with developmental disorders such as ASD, ADHD, or learning disorder. Though there were differences in diagnosis, those differences were not predictive of outcome. A regression analysis indicated that the clinical site did not predict changes in power per session or coherence score per session.

### Apparatus and Materials

#### EEG Data Collection

The EEG assessment was conducted during eyes-closed and eyes-open resting conditions while subjects were seated on a reclining chair. Data collected formed the basis for evaluating change in coherence scores for the current study. An Electrocap International recording cap containing 19 sensors was attached to participants’ scalps to collect data. The caps provided frontal reference, prefrontal ground, and linked ears.

Thatcher et al. (2003) assessed and confirmed the reliability and validity of QEEG. To obtain QEEG data, EEG readings were recorded and digitized using the International 10/20 System (Jasper, 1958) of electrode placement and the Deymed Diagnostic (2004) TruScan 32 Acquisition EEG System with a sampling rate of 256 Hz. The sensitivity was set at 70 l V/cm, low frequency filter 0.1 Hz, high-frequency filter 100 Hz and 60-Hz notch filter. Common mode rejection ratio was 102 dB and isolation mode rejection ratio was 140 dB, comparable between both groups. Impedance levels were set at less than 5 kOhms. QEEG provides a mathematical analysis between individual EEG readings and normative samples matched for age and gender to identify inconsistencies in EEG neural functioning. Participant QEEG data was collected before and after selected coherence-guided neurofeedback protocols.

#### Neurofeedback Equipment

This study used the EEG Software EEGer Training System (EEG Software, 2013) to provide coherence-guided EEG biofeedback training to participants. During training, “Grass Silver Disc 48” Electrodes with SafeLead protected terminals (Grass SafeLead, 2006) were placed on participants’ scalps to measure EEG activity. Participants viewed immediate feedback on the relative amplitude/threshold values of the signal in the form of visual and aural cues. Simple graphics in the form of computer games continuously presented the ratio of amplitude to threshold for each stream of data to provide visual feedback to participants. To provide aural feedback, whenever participants achieved a specific amplitude/coherence condition, they heard a pre-recorded sound file, a short quarter of a second beep, one or fewer times per every half second (EEG Software, 2013). Each participants’ neurofeedback protocol was designed to complement the findings of their individual, initial QEEG analysis.

#### Patient Protocol Development

For both training groups, the following procedures were employed to produce individualized patient protocol. Patient data is first artifacted manually through the NeuroRep software suite (Hudspeth, 1999). Epoch rejection was based on visual inspection and included the removal of artifact such as blinking or head movements. The EEG is then compared to normative databases matched for age. The databases for comparisons included: BrainDx (BrainDx, 2014), NeuroRep (Hudspeth, 1999), and Neuroguide (Applied Neuroscience Inc., 2017). Frequency band maps are then created and compared to a normative database. Database analyses are produced for absolute power, relative power, asymmetry, and coherence values. The use of NeuroRep for multivariate coherence analysis helps us to understand complex synchronization patterns. When one examines these images, relative regions of hyper- or hypo-coherences may be observed.

The primary investigator consulted with all clinics and developed and provided all patient protocols. Treatment was personalized for each individual on the basis of his or her QEEG findings for power and coherence. Areas that showed hypocoherence (too low coherence) or hypercoherence (too high coherence) were targeted. Protocol designs were different for each participant with the rewarding and inhibiting frequency matched to the frequencies showing the greatest problems of coherence and power. The sessions ran for 15–20 min in duration. The participants that made up the two channel coherence training group received on average 21.49 sessions of two channel coherence guided EEG coherence neurofeedback. The participants that comprised the four channel multivariate coherence training group received on average 13.65 sessions.

### Procedure

This study was a between-group experimental study. Participants were assigned to receive either the new four channel multivariate coherence training or traditional two channel coherence training. Participants’ neurofeedback protocol was designed based on the findings of their personal QEEG data, regardless of which experimental group they belonged. To develop participants’ treatment protocol, initial EEG data was individually compared to a normative database to establish baseline levels of coherence, absolute and relative power. The neurofeedback protocol was then designed to complement these findings. Participants’ coherence and power change scores were calculated from individual QEEG data both pre- and post-treatment. These values were compared to measure progress.

### Design and Analyses

The dependent variables explored change in coherence and power based on the findings of the NeuroRep software suite. The wCompare2 feature of NeuroRep was used to compare the initial and post treatment QEEGs. The wCompare2 feature shows a comparison value that represents a percentage change in coherence between two EEGs at each electrode across the delta, theta, alpha, and beta frequencies bands. Based on the protocol of each participant a regional change was calculated between the electrodes that were selected for the participant’s individualized protocol. Each value from the eyes open data stream was added across each frequency band for both inter and intra hemispheric changes. The resulting sum will represent the coherence change score. An adjusted coherence score was then calculated for those who had been assigned QPS deviation. Those who received QPS deviation have had their sign of their coherence score reversed, for example if the score was “−11” it would be changed to “11.”

Finally, the coherence score was broken down into a change per session score. This score was calculated because on average the participants in the two channel coherence group had around 21.49 sessions on average. Those in the four channel multivariate coherence training group participated in 13.65 sessions on average. The coherence score per session represented the coherence score divided by the number of neurofeedback sessions the participant had.

$$\begin{array}{c} \mathrm{Coherence Score}=\sum \left(\mathrm{inter}+\mathrm{intracoherence value}\right)\\ \mathrm{Coherence Score per Session}=\frac{\sum \left(\mathrm{inter}+\mathrm{inratcoherence values}\right)}{\mathrm{of sessions}} \end{array}$$

A separate measure within the wCompare2 feature in NeuroRep calculates a change in power from one recording to the next. This function of wCompare2 was used to create a power change score. The power change score was calculated using the max power percentage from both the eyes open and eyes closed condition and calculating their average. The power scores were also converted in a change in power per session score due to differences in average treatment sessions completed per treatment group. This value was calculated by dividing the power score by the number of sessions completed.

$$\begin{array}{c} \mathrm{Power Score}=\sum \left(\mathrm{eyes open}+\mathrm{eyes closed power}\%\right)\\ \mathrm{Power Score per Session}=\frac{\sum \left(\mathrm{eyes open}+\mathrm{eyes close power}\%\right)}{\mathrm{of sessions}} \end{array}$$

### Statistical Analysis

A Levene’s test of Homogeneity of Variances was used to compare variances in number of sessions across treatment types. Levene’s test determined that there was equal variance between number of sessions each group had thus a one-way ANOVA was selected to assess difference between the groups. The one-way ANOVA determined that the number of sessions per treatment did not have equal means. On average the participants in the two channel coherence group had an average of 21.49 sessions, while those in the four channel multivariate coherence training group participated in 13.65 sessions. Due to a difference in number of sessions per group, the power and coherence scores were give a “per session score” as described in Section “Design and Analyses.”

Statistical tests for the power per session scores, adjusted coherence per session score, and modality were chosen based on Levene’s test of homogeneity of variances. Our aim was to determine if there was a difference in means between the two groups. Levene’s test determined that between treatment groups, we could assume equal variances for power per session and modality. This led us to use a one-way ANOVA to assess difference in group means. Levene’s test determined that we could not assume equal variances for the adjusted coherence per session score, therefore we used the Mann–Whitney test where the base assumption is not equal variances.

An ANOVA test and Mann–Whitney test were used to test parameters across the measured groups. All *p*-values were less than 0.05 for measured results. Groups consisted of more than 40 participants each, so a high statistical power was observed. All statistical analysis was conducted using IBM SPSS Statistics 21.0 (IBM Corporation, 2012, Armonk, NY, United States).

A Kruskal–Wallis test was used to determine if there were differences in outcome scores for power per session and adjusted coherence per session across the different participating clinics. The Kruskal–Wallis was selected due to the known non-parametric nature of the clinic sites as determined by Levene’s test of homogeneity of variances.

## Results

No demographic variable, including medication, was significantly different between the groups. There were also no significant differences across treatment clinics on demographics and treatment clinic was not predictive of outcome. An independent samples Kruskal–Wallis test was conducted to compare the effects of treatment clinic on adjusted coherence per session scores and power per session scores. The changes in the adjusted coherence per session scores for the clinics were not significantly different (*p* = 0.202, *F* = 0.862, *df* = 9). Additionally, the changes in the power per session score were not significantly different (*p* = 0.293, *F* = 1.743, *df* = 9). Therefore, we believe that collapsing each clinic into its corresponding treatment group is reasonable. The type of coherence treatment participants engaged in was the only variable connected to differences in treatment outcome suggesting that treatment type was the only critical factor or variable.

**FIGURE 4:**
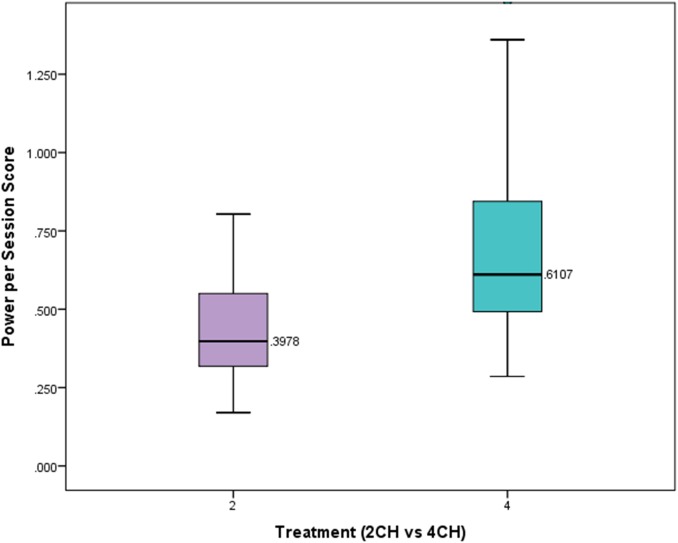
Box plot showing per session change in power for experimental and comparison group. There is a statistically significant difference (*p* < 0.001).

We hypothesized that participants who received four channel multivariate coherence neurofeedback training would show greater degrees of change than those receiving two channel coherence training. The number of sessions in each treatment group was significantly different. On average those receiving two channel treatment received 21.49 sessions. Those receiving four channel treatment had on average 13.65 sessions of treatment. A one-way between subjects ANOVA was conducted to compare the effects of treatment on power scores per session for those in either the four channel or two channel group. There was a statistically significant effect of treatment on the power scores per session (*p* = 0.00004, *F* = 17.801, *df* = 1). **Figure 4** presents a box plot of means and standard deviations comparing the effects of treatment on the two groups. This shows that the four channel group had power changes that were more than 50% greater than the two channel group.

**FIGURE 5:**
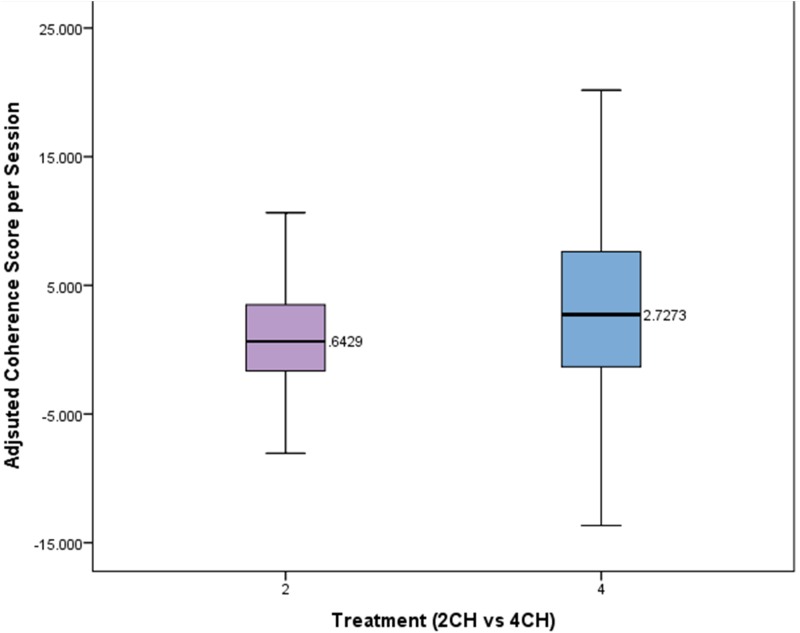
Box plot showing per session change in adjusted coherence for each treatment group. There is a statistically significant difference (*p* < 0.05).

A Mann–Whitney test was conducted to compare the effects of treatment type on adjusted coherences scores per session. The means and standard deviations of the adjusted coherence scores per session for the two channel and four channel groups are presented in **Figure 5**. The changes in coherence scores for the four channel treatment group were significantly greater (*Mdn* = 2.72) than for the two channel treatment group (*Mdn* = 0.64, *U* = 2741, *p* = 0.026). The four channel group showed coherence changes that were more than four times that of the two channel group per session.

**FIGURE 6:**
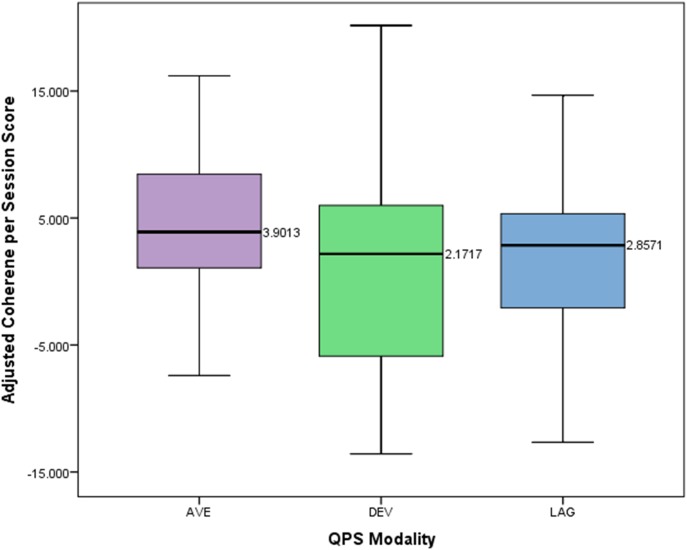
Box plot showing difference in adjusted coherence per session score across different QPS modalities.

A one-way between subjects ANOVA was conducted to compare the effects of modality of four channel multivariate coherence treatment on adjusted coherence scores per session for those in either the average, lag, or deviation group. The means and standard deviations of the AVE, DEV, and LAG groups are presented in **Figure 6**. There was not a statistically significant effect of treatment modality on the adjusted coherence scores per session (*p* = 0.397, *F* = 0.931, *df* = 2).

## Discussion

The breadth of neuropsychiatric problems calls for accessible and effective modes of treatment. The use of neuromodulation as treatment for neuropsychiatric problems attributed to brain dysfunction has begun to show efficacy in clinical populations. While the use of neurofeedback training is not new, recent advancements in technology may increase the availability and efficiency of this type of treatment. EEG based neurofeedback can offer a solution to the impracticalities included with the use of other forms of neuromodulation, such as fMRI, due to their high expense and lack of accessibility to many populations. This is important when the breadth of neuropsychiatric problems is considered. Recent sophistication in EEG analyses raises the likelihood that this technology may be used as an effective form of intervention.

The current study used an experimental design to assess the efficacy of a new form of neurofeedback training, four channel multivariate coherence training. A comparison group of participants received the standard two channel coherence neurofeedback therapy, while the experimental group received our model of four channel multivariate coherence neurofeedback therapy. Participants who received the new four channel multivariate coherence training improved their coherence and power scores more and over fewer sessions relative to those in the two channel coherence training group. Advancing to four channels and calculating coherence metrics in a multivariate fashion led to greater changes in power, by more than 50%, and coherence, by more than 400%. This was shown to be the case across multiple clinical settings and various clinical conditions in that clinical site was not predictive of outcome as indicated by a regression analysis.

Utilizing such a methodology hypothetically may enhance the efficacy of simpler versions of EEG neurofeedback as they are used currently. Single and two channel neurofeedback (EEG) has been shown to lead to significant improvements in functioning across clinical conditions such as ADHD (Arns et al., 2014), learning disabilities (Coben et al., 2015), ASD (Coben, 2013), and traumatic brain injuries (Bennett et al., 2017). Two channel coherence training has previously been implemented with demonstrated efficacy (Thornton, 2000; Walker et al., 2002; Thornton and Carmody, 2005; Walker, 2008; Mottaz et al., 2015). In fact, Coben and Myers (2010) have shown this approach to have greater efficacy than single channel training. However, this is the first time four channel multivariate training has been implemented and tested. Two possible reasons exist as to why this approach may lead to greater changes in the underlying neurophysiology of such difficulties. First, we used a larger number of electrodes which enables greater coverage of brain space. While it is possible that this alone may explain these findings, simply using more electrodes has not necessarily led to increases in clinical outcome (i.e., Hammer et al., 2011; Liechti et al., 2012).

We would propose that the differential effects, then, are more likely related to the increase in statistical sophistication in what is actually fed back to the subject. The multivariate nature of how coherence is calculated may come closer to the brain signal itself and thus make it easier for the subject to change. Traditionally and historically EEG coherence estimates have arisen from cross correlations between pairs of electrodes (Bendat and Piersol, 1980); the same is true to coherence training up to this point in time. While this approach has been commonly used in the past, there are certain limitations in its application and accuracy. Confounds in the accuracy of pairwise coherence measurements include inter-electrode distance, volume conduction and the inability to capture the three dimensional notion of brain activity (Nunez, 1994; Nunez and Srivinasan, 2006). It has further been observed that multivariate strategies to assess coherence metrics are more accurate and effective than their pairwise counterparts (Kus et al., 2004; Barry et al., 2005; Pollonini et al., 2010). This enhanced accuracy may translate into greater efficacy.

In this light it should be noted that the statistical sophistication of this multivariate approach may be enhanced. Sporns (2011) discussed effective connectivity as describing the network of causal effects between neural elements, which may be inferred through time series analyses, statistical modeling or experimental perturbations. These approaches enable us to see causal and reciprocal effects which are just not possible with pairwise coherences (Coben et al., 2014a). Future enhancements in this direction may lead to further increases in neurofeedback efficacy. Future researchers might also consider the use of event related potentials (ERPs) to help direct neurofeedback. Past research by Sokhadaze et al. (2018) has shown that monitoring ERP activity as a functional outcome of neuromodulation can be a powerful tool.

Of course, these findings are preliminary and should be replicated. Controlled research is suggested in applying this neurofeedback approach to clinical populations to investigate efficacy.

Faster treatment effects of the new four channel NFB introduces potential cautions that should be considered when applying this technique. More rapid results may require greater oversight therefore closer review of patient progress may be necessary. Those who are providing four channel multivariate coherence training should have higher levels of experience and training. Additionally, the NFB should be guided by QEEG and coherence analysis as indicated above. The set-up could take up to 2 min longer than for two channel bivariate coherence training, but processing time remains the same. There is no delay in the feedback for four channel multivariate coherence training. Additionally, temporal resolution is enhanced, rather than compromised.

An additional limitation of this study is that it does not address the relationship between the results and changes in participants’ symptoms. This was a preliminary study to investigate whether four channel multivariate coherence training produced a greater effect size than two channel bivariate coherence training. Further research is necessary to investigate the implications of these greater effect sizes for the treatment of clinical populations.

## Ethics Statement

This study was carried out in accordance with the recommendations of “Western Institutional Review Board”; with written informed consent from all subjects. All subjects gave written informed consent in accordance with the Declaration of Helsinki. The protocol was approved by the “ethics committee above.”

## Author Contributions

RC and HL contributed to the conception and design of the study. HL designed the A_PSync mode for neurofeedback in EEGer. RC and MM organized the database and performed the statistical analysis. MM wrote the first draft of the manuscripts. All authors wrote sections of the manuscript and contributed to manuscript revision, read and approved the submitted version.

## Conflict of Interest Statement

HL designed the A_PSync mode for neurofeedback in EEGer and was not involved in data collection or statistical analyses. RC provided analysis of QEEG data and protocol development for all sites, but was not involved in any way in running the neurofeedback sessions or collecting any outcome data. The remaining authors declare that the research was conducted in the absence of any commercial or financial relationships that could be construed as a potential conflict of interest.
